# Anthropogenic pollutants’ threats to deep-sea ecosystems: Research challenges and technological frontiers

**DOI:** 10.1016/j.eehl.2026.100271

**Published:** 2026-08-03

**Authors:** Rui Hou, Jing-Chun Feng, Si Zhang

**Affiliations:** a Institute of Smart Ocean Science and Engineering, School of Ecology, Environment and Ocean, Guangdong University of Technology, Guangzhou, 510006, China; b Guangdong Basic Research Center of Excellence for Ecological Security and Green Development, School of Ecology, Environment and Ocean, Guangdong University of Technology, Guangzhou, 510006, China; c Southern Marine Science and Engineering Guangdong Laboratory (Guangzhou), Guangzhou, 511458, China

**Prior tothe full recognition of its complexity, deep-sea environments have already been affected by human activities. Moving beyond descriptive and observation-bound monitoring, this*****Comment*****proposes an integrative framework that synthesizes pollutant dynamics with deep-sea hydro-biogeochemistry, extreme-environment ecotoxicology, and advanced*****in situ*****ecological monitoring. Transformative advances in integrated observation and simulation technologies will shift deep-sea pollution research from descriptive assessment to proactive forecasting, offering the critical evidence base required to conserve the ocean’s largest and most vulnerable realm.**

The deep sea, encompassing ocean depths below 200 m, represents more than 95% of the ocean’s volume and hosts a spectacular variety of ecosystems, including continental shelves, abyssal plains, seamounts, cold seeps, hydrothermal vents, and hadal trenches. Despite its remoteness, anthropogenic pollutants have permeated even the most inaccessible regions. Plastics, persistent organic pollutants (POPs), heavy metals, per/polyfluoroalkyl substances (PFASs), flame retardants, and antibiotic resistance genes (ARGs) are frequently detected in deep-sea water and sediments [1,2]. Particularly notable is the significant presence of pollutants in hadal trenches (e.g., chlorinated paraffin concentrations up to 14,000 ng/g dw at depths > 10,000 m [3]), highlighting the pervasive impacts of human activity. However, the deep sea remains one of the least studied environments, where technological and methodological challenges pose a major barrier to understanding the pollutant fates, toxicological mechanisms, and overall risks to deep-sea ecosystems.

## 1 Complex sources, hidden journeys, and unexpected fluxes of deep-sea pollutants

Terrestrial pollutants enter deep seas mainly through three pathways: 1) the biological pump, through which particulate organic carbon (POC) adsorbs contaminants and sinks vertically; 2) the physical pump, which is driven by thermohaline circulation and downwelling currents; and 3) the active biological pump, whereby zooplankton and micronekton perform diel vertical migrations (DVMs), feeding in surface waters and actively transporting carbon and associated contaminants to depth via respiration, excretion, and mortality within a 24-h cycle [4]. Notably, microplastics can also act as key carriers for hydrophobic pollutants, exhibiting greater persistence than POC (with the ratio of microplastic carbon to POC increasing from 0.1% at 30 m below the seafloor to 5% at 2000 m) [5].

Additionally, petroleum drilling and seabed infrastructure create localized pollutant hotspots, such as hydrocarbons, heavy metals, and radioactive materials. As the deep seabed harbors vast amounts of metals in the form of polymetallic nodules (PMNs), polymetallic sulfides, and cobalt-rich crusts, deep-sea mining has recently become a significant issue in marine sustainability regarding the risks of the additional release of heavy metals and other pollutants [6]. All types of deep-sea mining can generate sediment plumes, increase the bioavailability of toxic metals, and smother organisms [6,7]. However, the specific toxic metals involved vary among different mineral deposits, which may lead to different environmental risks.

Few studies have quantified the flux of pollutants into the deep sea, yet the findings are striking. An estimated 75% of cumulative polychlorinated biphenyls (PCBs) releases are buried in deep-sea sediments [8], while 99% of marine plastics eventually settle in the deep sea [1]. Semiempirical models indicate the global settling flux of PAHs via biological pumps is 8 Mg/year [9]. Therefore, further research is essential to provide critical insights into the sources, transformation mechanisms, and coupled transport processes of pollutants, thereby advancing our understanding of source–sink dynamics in deep-sea environments.

## 2 Impacts of anthropogenic pollutants on deep-sea fauna and ecosystems

The majority of pollutants entering deep-sea ecosystems exhibit high persistence and bioaccumulation potential, inevitably causing chronic exposure to local ecosystems. Several μg/g levels (lipid weights) of PCBs and polybrominated diphenyl ethers (PBDEs) have been found in amphipods collected from continental rises to hadal trenches (>6000 m) [10]. Furthermore, chronic exposure to PCBs is linked to long-term population declines in top marine predators and may also impact environmental adaptation and evolution of deep-sea fishes [11]. Particularly concerning are the adverse impacts on deep-sea biodiversity hotspots. Chronic pollutant exposure or even biomagnification in extreme deep-sea ecosystems (e.g., cold seeps, hydrothermal vents, cold-water coral reefs) triggers severe disruptions in the growth, development, and reproductive fitness of inherently sensitive deep-sea biota [10,12].

In addition, anthropogenic pollutants can critically affect biogeochemical cycling and nutrient functions in the deep sea. The sequestration of microplastics in the seafloor leads to iron accumulation on particle surfaces, promoting the anaerobic oxidation of methane and influencing methane migration in cold seeps [13]. The microbial metabolism of halogenated organic pollutants may also be related to the key energy-producing pathway for the deep-sea biosphere. It is important to consider such coupled processes with carbon, nitrogen, and nutrient cycling in deep seas when determining the adverse effects of pollutants on deep-sea ecosystems.

## 3 Key research gaps in deep-sea pollution in the context of unique environmental characteristics

The topographic and environmental characteristics of seabeds significantly influence the occurrence patterns and environmental behaviors of anthropogenic pollutants. The V-shaped topography of hadal trenches and their associated tectonic activities may establish them as sinks for sediments and organic matter, whereas unique topographies such as continental slopes and seamounts can hinder pollutant retention. Furthermore, spatial variations in temperature, the complete absence of sunlight, differences in redox potential, and the intensity of bioturbation collectively shape distinct biotic and abiotic pollutant transformation pathways across deep-sea environments. It is also noteworthy that human development activities in certain deep-sea environments may remobilize geogenic or historically sequestered pollutants, to which more attention should be paid.

The relatively high hydrostatic pressure, low temperatures, oligotrophic conditions, slow metabolic rates, and low biomass of deep-sea ecosystems may also lead to distinctive ecotoxicological effects and underlying mechanisms. Hydrostatic pressure affects both chemical reaction kinetics and physiological functions in deep-sea organisms, potentially exerting amplified ecological impacts [12]. In oligotrophic deep-sea ecosystems dependent on “marine snow (transported via biological pumps)” for energy supply, co-transported pollutants potentially exert synergistic effects on marine biota. Chemosynthetic ecosystems such as cold seeps and hydrothermal vents, which are persistently exposed to geogenic toxic compounds (e.g., hydrogen sulfide, and metals), may exhibit elevated tolerance to these pollutants via long-term adaptations. The unique species traits, ecosystem structure, trophic dynamics, and microbial activities in these chemosynthetic systems suggest that pollutant-response mechanisms in such environments may differ from those in photosynthetic systems [14].

**Fig. 1:**
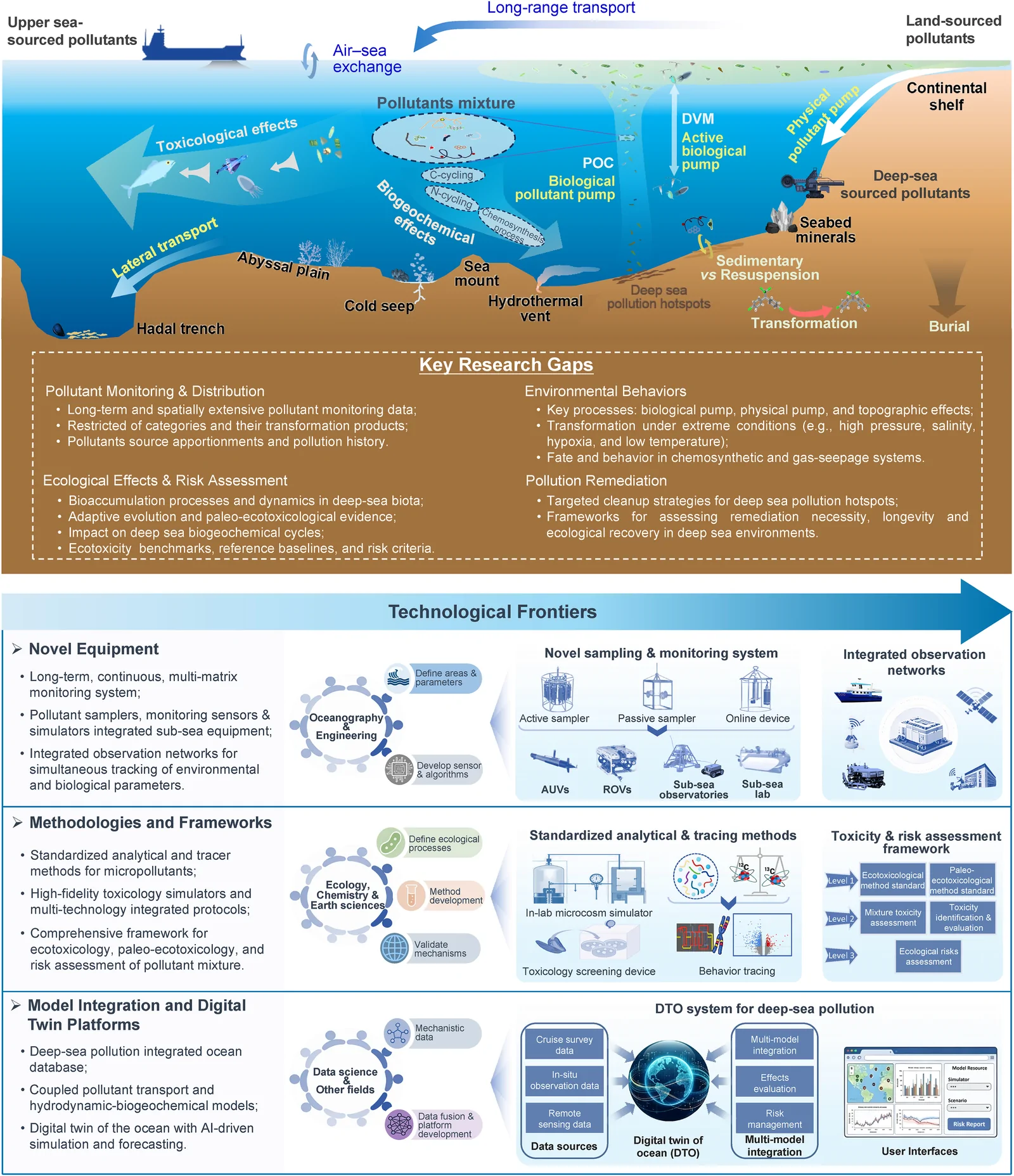
Critical research gaps and key technological frontiers toward a comprehensive “source—transport and transformation—ecological impacts” understanding of deep-sea pollution.

While scattered studies exist on the interactions of emerging pollutants with deep-sea ecosystems and biogeochemistry, critical gaps remain in establishing a comprehensive knowledge framework of four areas (Fig. 1): 1) adequate data on the pollutant sources, spatiotemporal distributions, and influencing factors across deep-sea environments; 2) key mechanisms governing pollutant transport and transformation, including biological, chemical, hydrodynamic, and bathymetric processes; 3) bioaccumulation pathways and toxicological responses within diverse deep-sea ecosystems and the long-term adaptive evolution of organisms to pollutants; and 4) remediation strategies for deep-sea pollution hotspots to advance the vision of a healthy and resilient ocean.

## 4 Technological frontiers toward integrated monitoring and predictive science

Deep-sea research depends critically on technology and infrastructure [15]. In stark contrast to existing research, which often focuses on single pollutants, isolated processes, or retrospective assessments, this work proposes an integrative framework that combines diverse equipment and technologies to deliver a mechanistically informed platform with predictive capability (Fig. 1).

1. Novel equipment and network. To overcome the limitations of traditional, cruise-based methods, which are often instantaneous, labor-intensive, and costly, novel equipment enabling long-term, continuous, multi-matrix monitoring and sampling is essential. Key development strategies include passive sampling technologies and *in situ* analytical platforms (e.g., electronic, spectroscopic, chromatographic, and mass spectrometric sensors) for next-generation investigations. Ultimately, these samplers, sensors, and simulators should be integrated into existing and future platforms such as remotely operated vehicles (ROVs), autonomous underwater vehicles (AUVs), human-occupied vehicles (HOVs), cabled observatories with docked crawlers, and future deep-sea submergence laboratories. Such integrated observation networks enable remote, long-term, and simultaneous tracking of pollutant fluxes and biological responses.
2. Advanced methodologies and frameworks. Standardized targeted and non-targeted analytical methods are required to detect complex mixtures of micropollutants (at trace or ultra-trace levels) across diverse deep-sea environments. Source and behavior tracing methods should be enhanced by incorporating isotopic and biomarker analyses; for instance, compound-specific stable isotope analysis of sediment cores can reconstruct the depositional history of organic pollutants. Furthermore, the development of high-fidelity deep-sea simulators and standardized protocols is essential to replicate extreme conditions (e.g., high pressure, low temperature, and oligotrophy) and enable high-throughput toxicological testing of individual and mixed pollutants using deep-sea model organisms. Multi-endpoint toxicity data, multi-omics on field-sampled biota, and biodiversity metrics derived from eDNA and optoacoustic imaging should be combined to identify key ecological indicators and early-warning signals. Finally, a comprehensive ecotoxicological assessment framework is needed to clarify causal mechanisms linking pollutant exposure to ecological impacts and to identify major environmental covariates.
3. Model integration and digital twin platforms. Mechanistic understanding requires coupling pollutant data and transport-transformation models with deep-sea hydrodynamic-biogeochemical models. With respect to data integration, deep-sea pollutant monitoring remains inadequately incorporated into established ocean databases and investigation networks. Aligned with emerging Digital Twin of the Ocean (DTO) strategies, an integrated technological framework should be developed that utilizes advanced instrumentation, big data analytics, and artificial intelligence techniques to enable comprehensive monitoring, simulation, and assessment of emerging marine pollutants. This DTO-enabled approach would close the loop from observation to prediction, enabling “what-if” scenario simulations for proactive ecosystem management.

Realizing this predictive framework demands interdisciplinary collaboration across oceanography, chemistry, biogeochemistry, ecology, microbiology, and engineering. Transformative advances in sampling and simulation technologies will herald an unprecedented exploration era of deep-sea pollution. These breakthroughs will catalyze novel research paradigms, elucidating interactions within the anthroposphere–deep-sea–biosphere nexus to overcome the research challenges in deep-sea pollution.

## CRediT authorship contribution statement

**Rui Hou:** Conceptualization, Funding acquisition, Validation, Writing – original draft. **Jing-Chun Feng:** Conceptualization, Formal analysis, Funding acquisition, Writing – original draft, Writing – review & editing. **Si Zhang:** Project administration, Resources, Writing – review & editing.

## Declaration of competing interests

The authors have no other relevant affiliations or financial involvement with any organization or entity with a financial interest in or financial conflict with the subject matter or materials discussed in the manuscript apart from those disclosed.
